# Mycelium-Composite Materials—A Promising Alternative to Plastics?

**DOI:** 10.3390/jof9020210

**Published:** 2023-02-06

**Authors:** Tiberius Balaeș, Bianca-Mihaela Radu, Cătălin Tănase

**Affiliations:** Department of Biology, Faculty of Biology, Alexandru Ioan Cuza University of Iași, 700505 Iași, Romania

**Keywords:** mycelial composites, biodegradable materials, fungal cultures, *Abortiporus biennis*

## Abstract

Plastic waste inefficiently recycled poses a major environmental concern attracting attention from both civil society and decision makers. Counteracting the phenomenon is an important challenge today. New possibilities are being explored to find alternatives to plastics, and one of them refers to mycelium-composite materials (MCM). Our study aimed at investigating the possibility of using wood and litter inhabiting basidiomycetes, an underexplored group of fungi that grow fast and create strong mycelial mats, to produce biodegradable materials with valuable properties, using cheap by-products as a substrate for growth. Seventy-five strains have been tested for their ability to grow on low-nutrient media and to form compact mycelial mats. Eight strains were selected further for evaluation on several raw substrates for producing in vitro myco-composites. The physico-mechanical properties of these materials, such as firmness, elasticity and impermeability, were analyzed. *Abortiporus biennis* RECOSOL73 was selected to obtain, at the laboratory scale, a real biodegradable product. Our results suggest that the strain used is a promising candidate with real possibilities for scalability. Finally, corroborating our results with scientific available data, discussions are being made over the feasibility of such technology, cost-effectiveness, scalability, availability of raw materials and, not least, where future studies should be directed to.

## 1. Introduction

Non-biodegradable waste represents a major environmental concern that has attracted attention from governmental bodies, decision makers and civil society. New policies are made to counteract this phenomenon, such as banning single-use plastic items [1] and encouraging the production of new biodegradable materials. Enormous amounts of plastic waste are accumulating in the environment, especially in the planetary ocean, posing a great threat to biodiversity [2]. Global production estimations vary; according to Aiduang and co-workers [3], 358 million metric tons of plastic were produced in 2018. Of the 2.5 billion tons of waste generated in 2022, nearly half are packaging materials [4]. Therefore, searching of alternative biodegradable materials has become a major challenge.

Among the new possibilities explored, the production of alternative fungal-based materials or mycelium composite materials (MCMs) has become a concept of much interest in recent years. Some progress has been made, and even commercial products are being currently marketed [2], with 36 fungal species named in patents filled [5]. However, the field is still in its infancy, and many questions remain unanswered. The subject has gained interest in the past 5 years [5], with a strong increase of research in the area. Several authors have reviewed literature in the field [6,7,8,9,10,11,12,13,14,15,16]. Numerous studies have been directed to the possible use of such materials, especially in the construction sector for thermal insulation [14,17,18,19,20], acoustic absorbers [21,22,23] or bricks [24,25]. The possibility of mycelium to be used as the bonding agent for wood boards [26,27,28] or combined with clay for “self-healing” purposes, replacing cement-based materials [29], was also investigated. Some futuristic investigation was aimed at 3D printing [30,31,32] or at producing leather-like materials [33]. Other authors analyzed the possibility of using MCM for packaging [3].

Wood-degrading (WD) or litter-inhabiting (LI) fungi have unique properties related to both the ability to colonize and degrade lignocellulosic substrate and the production of an extended network of hyphae [34,35]. Decomposition of lignocellulosic substrate is achieved through various enzymes these organisms secrete (cellulase and ligninase enzyme systems) [36]. Fungi producing white rot wood is particularly important for the production of MCMs due to their substrate efficient use as sources of nutrients. The dimitic and trimitic hyphal systems of many WD and LI fungi [5,34,37] make them create very strong layers of mycelium and thus composites with increased resistance, but most studies for MCMs production used only a few species of WD basidiomycetes, especially those well known as cultivated edible and medicinal mushrooms [6,38,39,40,41,42,43,44,45,46,47], while many valuable species and genera from this group remain untested.

Our study aimed at investigating new species and genera for their possible use in the production of MCMs, starting by screening 75 strains for the necessary properties, such as a fast growth rhythm and strong mycelial network formation. Eight strains were further selected and grown on lignocellulosic substrates to produce MCMs that were later analyzed for their mechanical properties. *Abortiporus biennis* RECOSOL73 strain was chosen to produce a final MCM molded into a particular shape with strong hydrophobic properties, suggesting the possibility for application in packaging. Further investigations are required to optimize the production of such materials and new available substrates to be used.

## 2. Materials and Methods

### 2.1. Fungal Strains and Reagents

Seventy-five fungal strains (Table 1) were used from the Culture Collection of Fungal Research Laboratory, Faculty of Biology, Alexandru Ioan Cuza University of Iasi, previously isolated using dikaryotic mycelium from fruit bodies and characterized [48,49]. All the analytical grade reagents for preparation of the culture media were purchased from Merck (Darmstadt, Germany). Wheat bran was purchased from a local food shop, sawdust (spruce sawdust) from a timber factory, wheat straw from a local farm and coconut husk fibers from a pet shop.

### 2.2. Inoculum Preparation and Culture Conditions

Fresh cultures were obtained on agar medium containing, L-1: malt extract 15 g, glucose 10 g and agar 15 g, pH 5.5, incubated at 25 °C for 14 days in an aerated incubator Aqualytic (MicroBiotests Inc, Gent, Belgium) and used as source of inoculum. Liquid cultures were prepared by homogenizing a 9 cm diameter solid culture with a Heidolph SilentCrusher M (Heidelph Instruments, Schwabach, Germany) (9000 rpm, 40 s) in 250 mL liquid media with the same composition as the solid one, except the agar, and incubated further for 7 days at 150 rpm, 25 °C. All the strains were primarily tested for the ability to grow on low nutrient media (L^−1^: glucose 8 g, peptone 0.5 g, (NH_4_)_2_SO_4_ 3 g, MgSO_4_ 0.5 g) using Petri dishes of 9 cm diameter, inoculated with agar plugs of 7 mm diameter cropped from the periphery of the colony, and incubated for 10 days at 25 °C. Organic substrates for MCMs were inoculated with 2 mL of liquid culture dispersed over the surface. All the experiments were performed in triplicate.

### 2.3. Evaluation of Growth Rhythm on Low-Nutrient Media

The ability to grow and produce compact mycelial mats for the 75 strains was evaluated by measuring the colonies diameters and through visual inspection under a stereomicroscope at 10–40×. Corroborating the gained data with previously obtained results [48,49], eight strains (marked with bold in Table 1) were further selected for MCMs production assessment. Three criteria were used for selection: (i) growth rhythm on low nutrient media (colony diameter); (ii) thickness and aspect of mycelium layer; (iii) firmness of mycelial mat formed (fragile or resistant when cutting with a loop).

### 2.4. Growth Substrates for MCM Production

**Figure 1:**
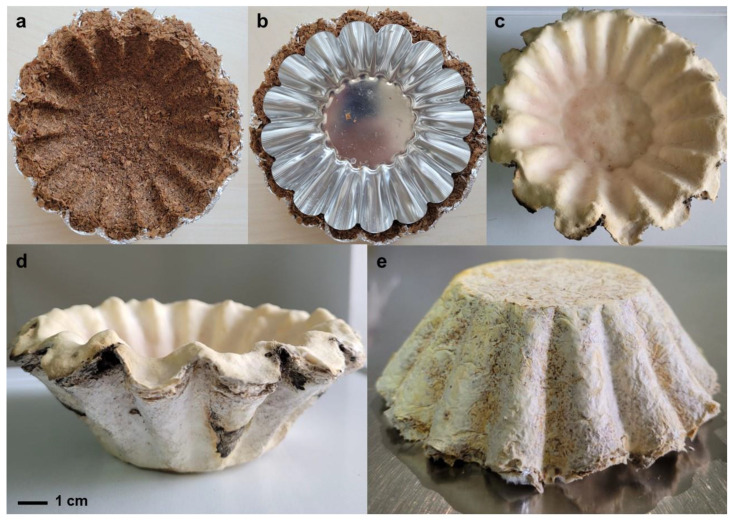
Obtaining the final material with *A. biennis* RECOSOL73 strain grown on wheat bran-sawdust substrate ((**a**–**c**)—molding the substrate; (**d**,**e**)—the final product).

Seven types of organic substrates were tested to produce MCMs: wheat bran, minced wheat straws; coconut husk fibers; broadleaves sawdust and mixtures of wheat bran + wheat straws, wheat bran + sawdust and wheat bran + coconut husk fibers at a ratio of 1:3. All the materials were soaked in distilled water overnight, the excess water drained, placed and manually compressed in Petri dishes of 9 cm diameter and sterilized through autoclaving at 121 °C. After inoculation, samples were placed at 25 °C for 12–32 days. For the final product, a mixture of wheat bran + sawdust was uniformly molded in a “baking tin” shape between two metal sheets (Figure 1) and sterilized by autoclaving at 121 °C. The dimensions were: 15 mm thickness (10 mm at wrinkles), 45 mm height, 80 mm diameter at bottom and 120 mm diameter at top. The materials were uniformly inoculated with 15 mL liquid cultures of *A. biennis* RECOSOL73 and incubated in a humid chamber at 25 °C for 3 days. After that period, to allow better oxygenation the cover the metal sheet was removed, and cultures incubated further for 27 days, assuring humidity.

### 2.5. Primary Assessment of MCMs

Materials obtained on Petri dishes were visually inspected during incubation period, dehydrated between filter paper sheets at room temperature and tested following the criteria: (i) colonization rate at the end of incubation through visual examination under stereomicroscope at 10–40× (100% colonization was considered when no area of substrate remained uncolonized); (ii) firmness of mycelium mat formed; (iii) permeability in water by placing droplets of water to the surface of material for 5 min (the material was considered low permeable if the water remained completely unabsorbed); (iv) resistance to fragmentation (brittle if materials break up very easily and do not oppose any sort of resistance; elastic if it bends without breaking up).

### 2.6. Evaluation of the Final Products

The final “baking tin” molded MCMs were analyzed for quality features: average density, long lasting impermeability and water uptake. Lasting impermeability was measured by placing water in the product on a sheet of filter paper for 76 h and verified periodically to assess if the water had penetrated the product. Water uptake was evaluated through immersion of product fragments in water for 76 h and weighing at 3, 24, 48 and 76 h to determine the quantity of water absorbed in the material. The thickness of the material was also measured at the same time points. Resistance to temperature was tested by placing fragments in an oven at 150 °C for 1 h and subsequently at 200 °C for 2 h. The structure was analyzed through microscopy.

### 2.7. Viability of the Mycelium

To verify if the mycelium contained in the material is neutralized after dehydration without thermal treatment, small fragments of the material taken aseptically from the inner part were placed on 2% MEA-containing Petri dishes and incubated for 7 days at 25 °C. After incubation, the inoculated Petri dishes were analyzed visually under stereomicroscope.

### 2.8. Optical and Electronic Microscopy (SEM)

All the cultures and obtained materials were analyzed through optical microscopy using a SZM2 stereomicroscope (Optika, Ponteranica. Italy) at 10–45× magnification and a contrast-phase trinocular microscope (Nikon Instruments, Tokyo, Japan) at 40–600× magnification. From the final MCMs, thin fragments were cut from both surfaces and from the inner part and examined through optical microscopy to reveal the network of hyphae developed around the solid particles. Additionally, similar samples were analyzed through SEM methos with a Vega II SBH, Tescan (Brno, Czech Republic). Prior to analysis, samples were dehydrated at room temperature and coated with a 30 nm layer of gold EMS 550X Sputter Coater, Electron Microscopy Sciences (Hatfield, PA, USA) and examined at an acceleration voltage of 30.00 kV.

### 2.9. Statistical Analysis

Experiments were performed in triplicate. Mechanical properties of final MCM were determined in ten replicates. Results were statistically analyzed with Dunnett’s multiple comparisons test, the data being presented as mean (*n* = 10) ± SEM. Data were analyzed with GraphPad Prim 9 software (GraphPad Software, Inc., La Jolla, CA, USA). Differences between groups were considered significant when *p* < 0.05.

## 3. Results

### 3.1. Low Nutrient Media Growth

A primary screening was involved, testing 75 strains of WD and LI fungi for their ability to grow on low nutrient media and production of material mats. Both the structure and aspects of mycelial mat varied among the tested strains (Table 1). Several strains fully covered Petri dishes in 10 days, but their mycelial network was different. Development of mycelium varied from yeast-like structure (*I. cuticularis* RECOSOL23) to rigid crusts (*P. badius* RECOSOL43) and to very floccose aerial mycelium, and even organized as rhizomorphic cords (*C. striatus* RECOSOL7). Additionally, some had very thin and tenuous structure, with lax hyphae developed at the surface of the media (*B. fumosa* RECOSOL90), being very fragile and easily breakable. Other strains produced a thick mycelium with numerous aerial hyphae and even conidiophores, yet very fragile (*L. sulphureus* RECOSOL81). Several strains developed thick and dense mycelium with numerous branched hyphae creating strong mats but had a slow growth rhythm. Although several strains grew fast and also many produced resistant mats (Table 1), not all these strains overlapped, and for this reason, only eight were selected for further studies.

The selected strains (marked with bold in the table) created a thick compact mycelium with skeletal and binding hyphae that gave resistance. In some cases, even small crusts were formed by cuticle cells. When observed at stereomicroscope, the hyphal network appeared either appressed and compact (*F. fomentarius* RECOSOL61); appressed to raised and wooly-felty (*D. tricolor* RECOSOL60, *L. arcularius* RECOSOL40 and *T. versicolor* RECOSOL94) or raised, forming a layer up to 3–5 μm thickness, cottony woolly floccose (*A. biennis* RECOSOL73, *B. adusta* RECOSOL20, *I. lacteus* RECOSOL25 and *P. ostreatus* RECOSOL159). Clamps connections were seen for *A. biennis*, *B. adusta*, *F. fomentarius*, *P. ostreatus* and *T. versicolor* and were absent for *D. tricolor*, *I. lacteus* and *L. arcularius*. Skeletal hyphae were also detected, and more abundant were recorded for *I. lacteus* and *P. ostreatus*. Abundant chlamydospores were observed in *A. biennis* cultures (Supplementary Figure S1) but also for other strains not selected (i.e., *G. resinaceum* RECOSOL17).

### 3.2. Primary Assessment of MCMs

Eight strains were further selected to produce MCMs and grown on 7 lignocellulosic substrates. The growth rhythm varied with the strain and the substrate used (Table 2), covering the plate in 12–32 days. Among the evaluated raw materials, coconut husk fiber was not efficiently colonized by mycelium of any strain tested.

Although several strains grew fast on different substrates, the mycelium development was poor in some cases, with a reduced colonization rate. Wheat bran appears to be easily used as source of nutrients by all strains.

**Figure 2:**
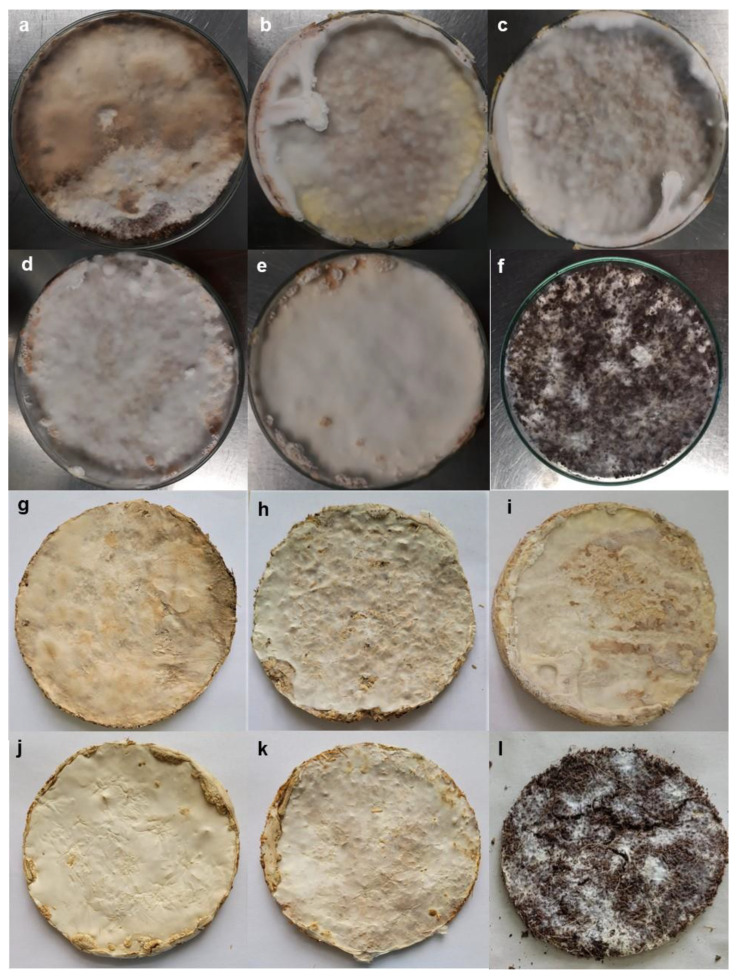
MCMs obtained with *A. biennis* RECOSOL73, a-f: mycelium growth after 3 weeks incubation on 9 cm diameter Petri dishes; (**g**–**i**): dried material ((**a**,**g**)—on wheat bran; (**b**,**h**)—on wheat straws; (**c**,**i**)—on sawdust; (**d**,**j**)—on wheat bran + wheat straws; (**e**,**k**)—on wheat bran + sawdust; (**f**,**i**)—on wheat bran + coconut husk fibers).

After incubation period, all the MCMs were removed from Petri dishes and dehydrated between filter paper sheets to conserve the shape. Their mechanical properties proved to be very different (Table 3) even in the case of the same substrate colonized by different strains. MCMs obtained with coconut husk fibers (either pure or in mixture) were excluded from these tests, as their quality was very poor (Figure 2), and they were very fragile and brittle. It appeared that the strain involved is influencing in a greater extent mechanical behavior than the substrate itself. *A. biennis* RECOSOL73 produced the most resistant materials and with a low permeability. MCMs obtained with wheat bran or mixtures containing wheat bran manifested better resistance and lower permeability, while the spruce sawdust was not efficiently colonized.

*A. biennis* RECOSOL73 created very compact MCM discs (Figure 2) with mycelium completely covering both surfaces in a strong compact layer. These discs were resistant and some of them manifested elasticity (Table 3).

Further on, due to the overall good quality, *A. biennis* strain was chosen to create the final MCM using sawdust mixed with wheat bran as reinforcing agent in a ratio of 3:1.

### 3.3. Evaluation of the Final Products

After the selection of the eight strains to inoculate seven different organic substrata, one strain has been chosen for production of MCM with a particular tin backing shape. After growth, the physical properties of the obtained material were tested concerning its lasting impermeability, water uptake after fragmentation, density and resistance to high temperature.

**Figure 3:**
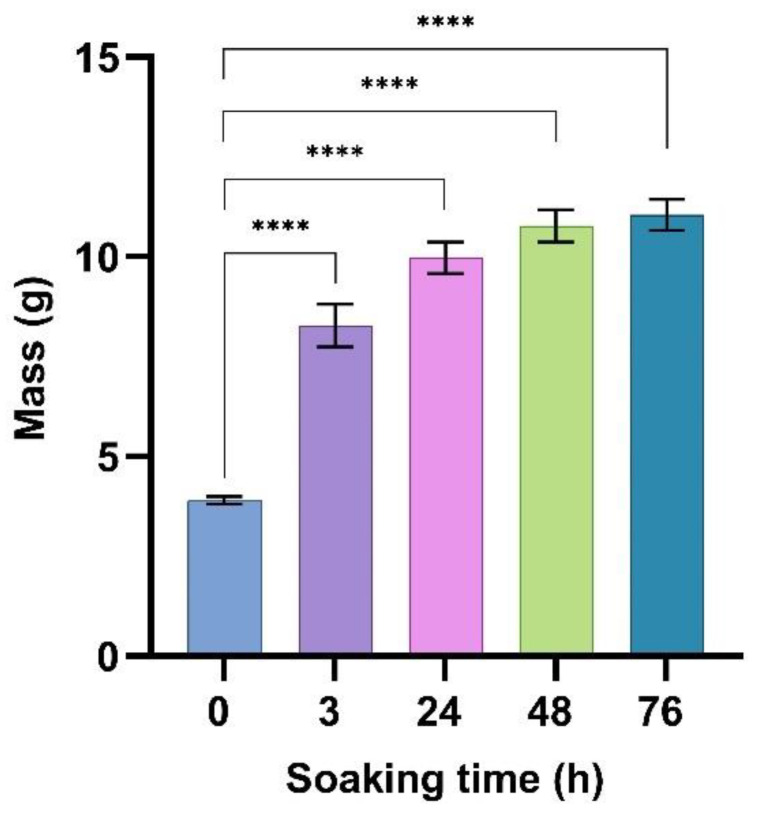
Mass increase during water uptake of material when soaked as cut fragments (**** significant differences for *p* < 0.0001).

The initial density of the material was 0.255 g per cm^3^, lower than other researchers reported [17]. When the integrity of the material was not affected, the mycelial mat, formed at surface, created a layer not permeable to water (even manifesting hydrophobicity) for 76 h, the inner part remaining dried. Disrupting the outer layer by fragmenting the material allowed the water to penetrate through the porous structure in the core, resulting in water uptake and increasing the mass up to 3 times after 76 h immersion compared with initial mass of the fragment (Figure 3). Thickness of the material was not affected by water uptake and the material was not swollen, meaning that most of water just filled the porous structure and to a lesser extent has been absorbed by solid particles of substrate. However, differences in the surface structure between the upper side and the bottom side have been observed, as the last one presented a less development of mycelial layer. This is due to keeping the metal sheet cover until the end of incubation, meaning that oxygen availability and space were reduced.

**Figure 4:**
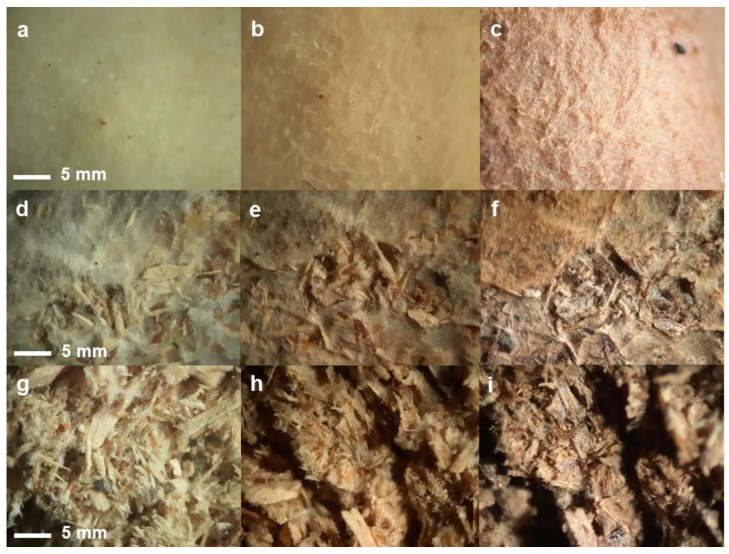
Analysis under stereomicroscope (15–35× magnification) of the material structure after exposure to high temperature ((**a**–**c**)—inner surface; (**d**–**f**)—outer surface; (**g**–**i**)—internal section; (**a**,**d**,**g**)—initial structure; (**b**,**e**,**h**)—after 1 h at 150 °C; (**c**,**f**,**i**)—after 2 h at 200 °C).

The obtained MCMs were evaluated for resistance at temperature. After placing it for 1 h at 150 °C in the oven, the material was visually inspected at stereomicroscope. While small changes in color were recorded (Figure 4), no significant other changes in mechanical properties were observed, except small gaps formed in the internal structure. Placing the material for 2 h at 200 °C resulted in a strong alteration of the structure, strong brown color, and fragility. Similar phenomena were observed by other authors [50]. The outer layer of mycelium remained resistant and flexible, easily detachable from the rest of the material.

### 3.4. Structure of the Material (Optical and Electronic Microscopy—SEM)

**Figure 5:**
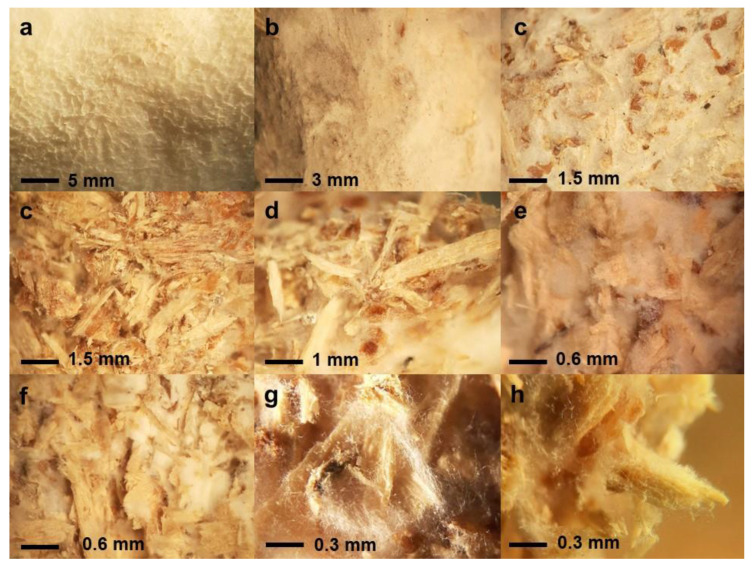
Structure of the MCM analyzed under stereomicroscope (20–40×): (**a**,**b**)—inner surface; (**c**)—outer surface; (**d**–**h**)—section).

When analyzed through microscopy, the structure appeared sandwich-like, with two strong layers of mycelium at both surfaces (Figure 5). The upper surface presented a thicker and more aerated layer compared with the bottom one, which appeared to be more compact and smoother, but thinner, due to direct contact with the metal sheet. Many anastomoses of hyphae were seen here.

At the interior, the mycelium was less developed, sawdust particles being easily visible across sections. However, around solid particles of sawdust numerous hyphae grew, binding these particles together.

**Figure 6:**
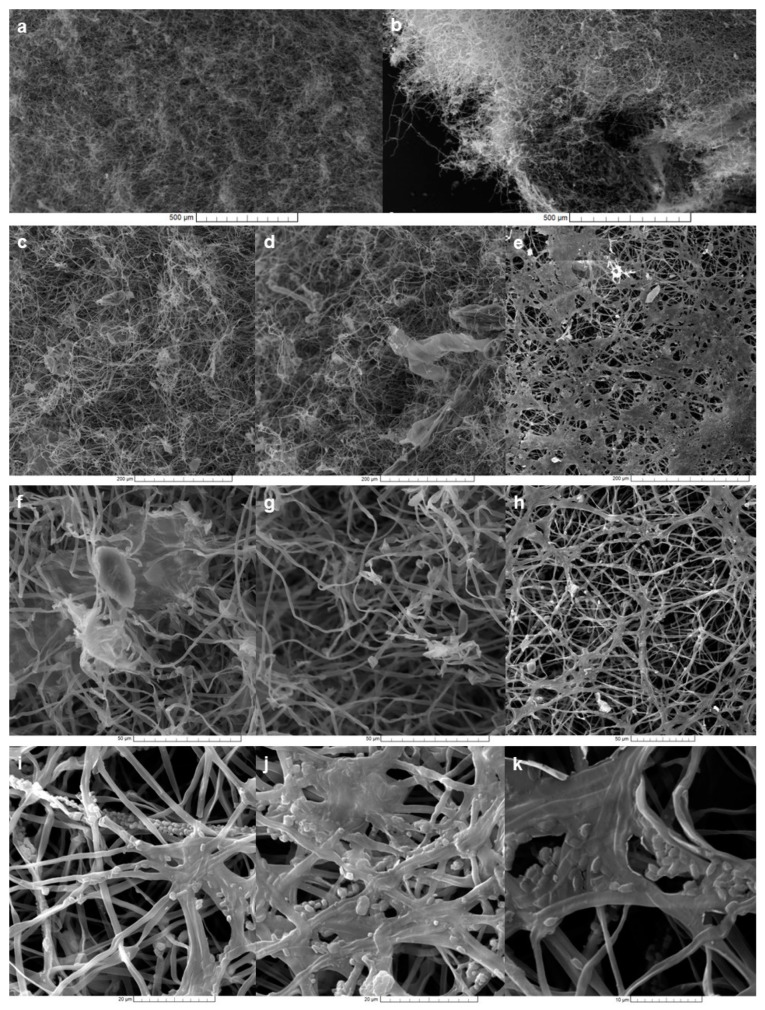
Surface structure of dried material seen under electron microscopy (SEM): (**a**,**c**,**d**,**f**,**g**)—upper surface; (**b**,**e**,**h**,**i**,**j**,**k**)—bottom surface.

Scanning electron microscopy was used to reveal in detail the MCM structure. At the upper surface, very branched hyphae forming a fluffy layer were observed (Figure 6), while the bottom one had many anastomoses created between hyphae, sometimes as plaque. Numerous chlamydospores were recorded here.

### 3.5. Viability of the Mycelium

Small fragments aseptically taken from the inner part of material were plated on malt extract (2%) agar to assess if the mycelium is still viable without heat inactivation. Colonies were formed, but their appearance might be generated by chlamydospores observed during microscopical examination. No clear evidence that hyphae forming the mycelium are still viable was observed.

## 4. Discussion

Fungal mycelium has unique properties obtained through a long-time evolution. Such features make filamentous fungi to be good partners for plants during mycorrhizas establishment, due to their substrate exploration ability [51]. These organisms have, also, a strong capacity to form large and complex networks of hyphae that bind solid particles together preventing soil erosion [52]. This last property of fungal mycelium, combined with ability to grow on various organic substrata make some saprotrophic species to be good candidates for production of biodegradable materials, as these fungi can use the organic matter for both nutrition and source of solid particle to bind.

Often, WD and LI fungi have ecological adaptations useful to produce MCMs such as antagonism properties [53], important for industrial scalability when considering raw materials that are highly impure and possibly heavily contaminated with microorganisms. This is more important if the contamination is with resistance spores (chlamydospores) that are difficult to inactivate.

Another important feature of these fungi is their ability to use nitrogen and phosphorus very efficiently from the substrates that are lacking these elements, such as many lignocellulosic wastes [54]. These properties of WD and LI fungi, make them suitable to grow on very diversified substrates.

Production of skeletal and binding hyphae (di- and trimitic hyphal systems) helps WD and LI fungi to create very strong mycelial networks [5,34,37], binding together different solid particles from the substrate they colonize.

In our study eight strains were tested on 7 raw lignocellulosic substrates. Some of the evaluated strains belong to species already known for MCM production, such as *T. versicolor*, *G. lucidum* and *P. ostreatus* [18,33,38,55], but they did not create the best material in our investigation. One possible explanation relies on their natural adaptation to grow on broadleaves wood, while we used spruce sawdust. Therefore, testing them on broadleaves sawdust might lead to different results.

For the selection of the eight strains, an additional criterion has been followed: to not pose environmental or human risks. The selected strains are being considered harmless and lacking pathogenicity towards humans and animals, or toxicity. *P. ostreatus* is an edible widely cultivated mushroom [33], *T. versicolor* is considered medicinal, while the other strains are saprotrophic wood-degrading species (except for *F. fomentarius*, which is sapro-parasite on broad leaves trees), common and with a wide distribution on the European continent [34,35,48,49]. Even more, it is supposed that after production of degradable myco-composite, the mycelium would be heat inactivated.

In our study, selected strains comprised both well investigated and less or un-investigated WD and LI fungi for MCM production, leading the way for new promising materials with valuable properties.

On the straw substrate, mycelium from different strains formed less resistant MCMs, and in many cases colonization was reduced, which might be explained by the aerial spaces between particles. If mechanically compressed, or grinded to small particles, mycelium would colonize it more efficiently. Wheat bran acted both as a good substrate itself and as a reinforcing agent, its small particle filling the gaps. However, cereals bran can hardly be considered nowadays as cheap and available resources. Other authors [56] reported increased mechanical properties of MCMs obtained with wheat bran, either pure or in mixtures.

The resistant MCM created by *A. biennis* RECOSOL73 mycelium on spruce sawdust is opening new perspectives as this type of sawdust is abundant in different areas dominated by coniferous woodlands.

Strong surface-hydrophobicity of the final MCM proven when material was not broken is a valuable property if such a material is considered for packaging fragile objects that are sensitive to humidity. However, water uptake when material is fragmented is important for biodegradation efficiency at the end of usage, through composting. The low density recorded, lower than other materials [17], make it even more suitable for packaging items production.

There were differences between the two surfaces as the mycelium stayed in direct contact with the metal cover at the external surface, resulting in less oxygen and space for development. A differentiated structure at the core compared with surface has been revealed by microscopy, with very strong layers of mycelium at surfaces conferring flexibility to some degree. Small particles of wheat bran filled the gaps in sawdust, helping the production of a more compact material.

The formation of chlamydospores during mycelium development is a drawback, these spores being produced to resist adverse conditions. For this reason, a higher temperature should be used for heat inactivation. However, there are chances that chlamydospores formation to be avoided if inoculation of substrate is achieved in a more even way, resulting in a shorter incubation time required. Chlamydospores are more often produced in old cultures that have been incubated for longer periods.

### Final Considerations for Scalability

Naturally, is difficult for biodegradable materials to compete with plastics when considering production costs. However, governmental support might contribute to entering the market for these products. Due to environmental hazard posed by accumulation of plastics residues both in marine and terrestrial ecosystems across the globe, many states on different continents and especially the European Union have imposed drastic measures to reduce plastic dumping [1]. Banning commercialization of some plastic products, such as single use plastic bags, is one of the strategies applied. At the same time, in the framework of Horizon Europa funding program, the developing circular economy is a major area to be achieved, meaning that both academia and industry have access to funding for R&D activities involving re-integration of by-products and residues in new technological processes [57]. These funding tools might offer good incentives for researchers to develop competitive technologies for transforming various types of wastes into biodegradable materials.

Some substrates, such as wheat bran, and in particular cases sawdust, cannot be considered anymore as affordable and easily available raw materials since they are currently being used in various processes and there is a high market demand for them, becoming scarcer. The most valuable raw resources should be those resulting from agri-food industry or from green zone maintenance in urban areas. Globally, there are large quantities of agricultural residues obtained each year, most of it consisting in lignocellulosic materials [58,59], therefore, such raw resources are highly available in some areas. A group of researchers [42] explored the idea of using a mixture of food waste, diaper waste and sawdust to produce formaldehyde-free boards.

There is not undoubtedly scientific evidence that fungal species that produce perennial (*F. fomentarius*) or annually, but resistant fruit-bodies (*Trametes* spp.) might be better at creating strong mycelial mats, but some might hypothesize that these species would develop more resistant mats since they produce skeletal hyphae—cells with thicker walls, with many branches, having a mechanical role in the formation of fruit-bodies [35]. This sort of hyphae appears in in vitro cultures [48,60]. On the contrary, species such as *Laetiporus sulphureus* do not produce skeletal hyphae and their fruit-bodies are also very fragile. In search of best candidates for myco-composites, researchers should direct their attention especially to these “resistant” fruit-bodies producing species. Literature available on the subject denotes that most researchers tested only a limited number of WD and LI fungi, many other species being unexplored, yet valuable properties might be discovered. Even more, most studies were focused only on resistance and density, but many other qualities of such materials might be useful and should be investigated.

Some researchers tried to assess the synergism between WD fungi and bacteria [61] or bacterial cellulose [62], but the synergism between different fungi, especially different WD and LI fungi has not yet been explored.

Another valuable feature that a fungal strain should have is the ability to grow on a wider organic substrate, to be easily applied where such diverse raw materials are available. Those species that grow on a limited range of substrates (such as monophagic fungi that develop only on a particular type of wood) are to be avoided.

A particular attention should be directed towards largely cultivated edible species (*Pleurotus* spp., *Lentinula edodes*, *Flammulina velutipes*) or medicinal cultivated mushrooms (G. lucidum) due to their wider usage, and therefore many fast-growing strains being available, and much knowledge accumulated concerning their nutrient requirements [63]. An additional advantage of using such strain for MCMs production arises from the possibility of using the spent mycelium-substrate resulting in mushroom cultivation as source of inoculum and thus reducing the operational costs. Even more, the spent substratum might become incorporated in the MCM. Another option is to produce at the same time MCM and edible mushrooms [13], decreasing the costs further. The global industry of mushroom cultivation is very large with a total estimation of 43 million tons annual production in 2018–2019 [63], resulting in bigger quantities of spent substrate. Among the cultivated mushrooms, a large share is represented by WD fungi such as *P. ostreatus* and *G. lucidum*, already proved to be useful for MCMs production [64].

The inoculation method is also important for a fast growth of mycelium. The more dispersed the inoculum, the more even the mycelial mat formed. Ideally, the substrate should be well mixed with fungal spores or propagules (mycelium fragments), however, when molding the material is difficult to achieve this without contamination. On an industrial scale might be even more difficult, but not impossible [31], depending on the substrate used, quantity and technology involved. Fungal species characterized by appearance of an anamorphic stage have the advantage of producing asexual spores easily recoverable that can be used for inoculation, compared to species generating only sterile mycelia in vitro, and thus requiring homogenization.

Another factor affecting the cost of production is the preparation of the inoculum, and therefore the medium used for it. The simpler and cheaper the medium (without special compounds, growth factors or expensive nutrients), the smaller are the final costs. For this reason, is important that selected strain grow on very cheap and simple substrate.

## 5. Conclusions

Eight strains of wood degrading fungi were evaluated for production of mycelium-composite materials, using various lignocellulosic substrates. *Abortiporus biennis* RECOSOL73 strain produced the most notable composites. Grown on a mixture of sawdust-wheat bran, developed a strongly hydrophobic at surface material, with a density as low as 0.255 g per cm^3^ and strong resistance, making it a good candidate for production of biodegradable packaging items. Further studies are required for optimization.

## Figures and Tables

**Table 1:** Fungal strains evaluated for ability to grow on low nutrient media at 25 °C for 10 days.

Nr.	Tested Fungal Strains	Average Colony Diameter (mm)	Mycelium Mat	Resistance
**1**	***Abortiporus biennis* RECOSOL73**	**90**	**compact, thick, fluffy at surface**	**resistant**
2	*Amaroostia stiptica* RECOSOL42	45	thin, fluffy	fragile
3	*Apioperdon pyriforme* RECOSOL27	28	tenuous	fragile
4	*Auricularia mesenterica* RECOSOL3	45	tenuous	fragile
**5**	***Bjerkandera adusta* RECOSOL20**	**90**	**very fluffy**	**resistant**
6	*B. fumosa* RECOSOL90	90	tenuous	fragile
7	*Cerioporus squamosus* RECOSOL64	70	very tenuous	fragile
8	*C. varius* RECOSOL101	40	tenuous	fragile
9	*Coprinellus micaceus* RECOSOL95	90	fluffy	fragile
10	*Coriolopsis gallica* RECOSOL6	90	tenuous	fragile
11	*Crepidotus applanatus* RECOSOL93	10	tenuous	fragile
12	*Crucibulum laeve* RECOSOL91	65	tenuous	fragile
13	*Cyathus striatus* RECOSOL7	65	tenuous, fluffy	fragile
14	*Daedalea quercina* RECOSOL83	65	tenuous	fragile
15	*Daedaleopsis confragosa* RECOSOL80	90	thin, fluffy	resistant
16	*D. tricolor* RECOSOL10	90	tenuous, fluffy	resistant
**17**	***D. tricolor* RECOSOL60**	**90**	**thick, fluffy**	**resistant**
18	*Flammula alnicola* RECOSOL35	20	tenuous	fragile
19	*Flammulina velutipes* RECOSOL11	45	very tenuous	fragile
**20**	***Fomes fomentarius* RECOSOL61**	**90**	**compact, thick**	**resistant**
21	*Fomitopsis pinicola* RECOSOL13	90	tenuous	fragile
22	*Ganoderma adspersum* RECOSOL14	88	compact	resistant
23	*G. applanatum* RECOSOL15	48	compact	resistant
24	*G. lucidum* RECOSOL16	47	compact	resistant
25	*G. resinaceum* RECOSOL17	90	tenuous, fluffy	resistant
26	*Gymnopilus junonius* RECOSOL18	46	compact, fluffy	fragile
27	*Gymnopus dryophilus* RECOSOL100	15	tenuous	fragile
28	*Hericium coralloides* RECOSOL55	85	tenuous, fluffy	fragile
29	*Heterobasidion annosum* RECOSOL107	45	tenuous	fragile
30	*Hymenopellis radicata* RECOSOL76	90	very tenuous	fragile
31	*Hypholoma fasciculare* RECOSOL21	20	tenuous	fragile
32	*H. lateritium* RECOSOL22	30	fluffy	fragile
33	*Inonotus cuticularis* RECOSOL23	15	very tenuous	fragile
34	*I. hispidus* RECOSOL24	55	compact, fluffy	resistant
**35**	***Irpex lacteus* RECOSOL25**	**90**	**compact, fluffy**	**resistant**
36	*I. lacteus* RECOSOL32	90	tenuous, fluffy	resistant
37	*Laetiporus sulphureus* RECOSOL81	90	fluffy	fragile
**38**	***Lentinus arcularius* RECOSOL40**	**90**	**compact, thick**	**resistant**
39	*L. substrictus* RECOSOL57	88	compact, thin	resistant
40	*L. tigrinus* RECOSOL70	87	compact, fluffy	resistant
41	*Lenzites betulinus* RECOSOL56	90	tenuous	fragile
42	*L. betulinus* RECOSOL36	90	tenuous, fluffy	fragile
43	*Leptoporus mollis* RECOSOL92	90	tenuous	fragile
44	*Megacollybia platyphylla* RECOSOL71	18	tenuous	fragile
45	*Meripilus giganteus* RECOSOL85	90	fluffy	fragile
46	*Mucidula mucida* RECOSOL86	63	compact, fluffy	fragile
47	*Mycetinis scorodonius* RECOSOL109	38	compact	fragile
48	*Panus neostrigosus* RECOSOL69	90	compact, fluffy	fragile
49	*Peniophora incarnata* RECOSOL29	90	tenuous	fragile
50	*P. quercina* RECOSOL30	85	compact, thin	fragile
51	*Phellinopsis conchata* RECOSOL31	70	fluffy	resistant
52	*Phellinus igniarius* RECOSOL33	57	compact, fluffy	resistant
53	*P. pomaceus* RECOSOL34	70	compact	resistant
54	*Phlebia tremellosa* RECOSOL28	14	very tenuous	fragile
55	*Pholiota aurivella* RECOSOL37	20	tenuous	fragile
56	*Picipes badius* RECOSOL43	12	compact	resistant
57	*P. melanopus* RECOSOL110	90	tenuous	fragile
58	*Pleurotus eryngii* RECOSOL105	40	tenuous	fragile
59	*P. ostreatus* RECOSOL111	90	fluffy	resistant
**60**	***P. ostreatus* RECOSOL159**	**90**	**compact, fluffy**	**resistant**
61	*Plicaturopsis crispa* RECOSOL39	51	tenuous	fragile
62	*Schizophyllum commune* RECOSOL77	90	fluffy	fragile
63	*Skeletocutis alutacea* RECOSOL45	47	tenuous	fragile
64	*Stereum hirsutum* RECOSOL78	90	compact, fluffy	fragile
65	*S. subtomentosum* RECOSOL89	90	tenuous	fragile
66	*Trametes gibbosa* RECOSOL47	90	tenuous	fragile
67	*T. gibbosa* RECOSOL59	90	fluffy	fragile
68	*T. hirsuta* RECOSOL65	87	compact, fluffy	resistant
69	*T. ochracea* RECOSOL88	88	compact, fluffy	resistant
70	*T. pubescens* RECOSOL49	88	tenuous	fragile
71	*T. pubescens* RECOSOL79	90	tenuous	fragile
72	*T. suaveolens* RECOSOL50	85	compact, thin	resistant
73	*T. trogii* RECOSOL104	87	fluffy, thick	resistant
**74**	***T. versicolor* RECOSOL94**	**90**	**compact, thick, fluffy**	**resistant**
75	*Xylobolus frustulatus* RECOSOL54	15	tenuous	fragile

* Bold marked the strains selected for further studies.

**Table 2:** Time required for colonization on different substrates (WB—wheat bran, WS—wheat straws, S—sawdust, CH—coconut husk fibers, WB+WS—wheat bran + wheat straws, WB+S—wheat bran + sawdust, WB+CH—wheat bran + coconut husk fibers).

Isolates	Substrates	Colonisation Time (Days)
*A. biennis* RECOSOL73	WB, WS, S, WB+WS, WB+S	20
	CH, WB+CH	32
*B. adusta* RECOSOL20	WB, WS, S, CH, WB+WS, WB+S, WB+CH	32
*D. tricolor* RECOSOL10	WB, WS, S, WB+WS, WB+S	20
	CH, WB+CH	32
*F. fomentarius* RECOSOL111	WB, WS, S, CH, WB+WS, WB+S, WB+CH	32
*I. lacteus* RECOSOL61	WB	12
	WS, S, WB+WS, WB+S	20
	CH, WB+CH	32
*P. ostreatus* RECOSOL32	WB, S, WB+S	20
	WS, WB+WS	32
	CH, WB+CH	12
*L. arcularius* RECOSOL40	WB, S, WB+S	20
	WS, WB+WS	32
	CH, WB+CH	12
*T. versicolor* RECOSOL94	WB	12
	WS, S, WB+WS, WB+S	20
	CH, WB+CH	32

**Table 3:** Properties of MCMs obtained with IL—*I. lacteus*; TV—*T. versicolor*; PO—*P. ostreatus*; LA—*L. arcularius*; FF—*F. fomentarius*; BA—*B. adusta*; DT—*D. tricolor* (Scm—substrate covered by mycelium, Perm.—permeability; WB—wheat bran, WS—wheat straws, S—sawdust, WB+WS—wheat bran + wheat straws, WB+S—wheat bran + sawdust).

Strain	Substrate	Scm (%)	Firmness	Resistance	Elasticity	Perm.	Strain
**AB**	**WB**	**100**	**compact**	**medium**	**elastic**	**low**	**AB**
**AB**	**WS**	**100**	**compact**	**low**	**brittle**	**low**	**AB**
**AB**	**S**	**100**	**compact**	**medium**	**elastic**	**low**	**AB**
**AB**	**WB+WS**	**100**	**compact**	**low**	**brittle**	**low**	**AB**
**AB**	**WB+S**	**100**	**compact**	**high**	**elastic**	**low**	**AB**
BA	WB	100	compact	low	elastic	low	BA
BA	WS	100	compact	low	brittle	low	BA
BA	S	90	compact	low	elastic	low	BA
BA	WB+WS	90	compact	low	brittle	low	BA
BA	WB+S	100	compact	low	brittle	low	BA
DT	WB	80	lax	low	brittle	low	DT
DT	WS	60	lax	low	brittle	high	DT
DT	S	80	compact	low	brittle	low	DT
DT	WB+WS	70	compact	low	brittle	low	DT
DT	WB+S	75	compact	medium	elastic	low	DT
FF	WB	90	compact	low	elastic	low	FF
FF	WS	80	compact	low	elastic	high	FF
FF	S	50	lax	low	elastic	low	FF
FF	WB+WS	90	compact	low	elastic	low	FF
FF	WB+S	70	compact	low	elastic	low	FF
IL	WB	98	compact	high	elastic	low	IL
IL	WS	60	lax	medium	elastic	high	IL
IL	S	40	lax	low	brittle	low	IL
IL	WB+WS	100	compact	low	brittle	low	IL
IL	WB+S	60	lax	low	brittle	low	IL
LA	WB	100	compact	high	elastic	low	LA
LA	WS	80	lax	low	brittle	low	LA
LA	S	20	lax	low	brittle	high	LA
LA	WB+WS	90	lax	low	brittle	low	LA
LA	WB+S	100	compact	high	elastic	low	LA
PO	WB	85	compact	low	elastic	high	PO
PO	WS	40	lax	low	brittle	high	PO
PO	S	20	lax	low	brittle	low	PO
PO	WB+WS	50	lax	low	brittle	high	PO
PO	WB+S	75	compact	low	brittle	high	PO
TV	WB	85	compact	medium	elastic	low	TV
TV	WS	10	lax	low	brittle	high	TV
TV	S	15	lax	low	brittle	high	TV
TV	WB+WS	85	lax	low	brittle	low	TV
TV	WB+S	15	lax	low	brittle	low	TV

* Bold marked the most active strain.

## Data Availability

Tested strains can be made available upon reasonable request.

## Supplementary Materials

The following supporting information can be downloaded at: https://www.mdpi.com/article/10.3390/jof9020210/s1, Figure S1: mycelium mat features of selected strains seen at stereomicroscope (35× magnification; a—*A*. *biennis*, b—*B. adusta*, c—*D. tricolor*, d—*F. fomentarius*, e—*I. lacteus*, f—*L. arcularius*, g—*P. ostreatus*, f—*T. versicolor*, g—chlamydospores of *A. biennis*).
