# Supplementary figures and images for: Mycelium-Composite Materials—A Promising Alternative to Plastics?
Source: J Fungi (Basel). 2023 Feb 6;9(2):210. doi: 10.3390/jof9020210 (PMC9965147; doi:10.3390/jof9020210)

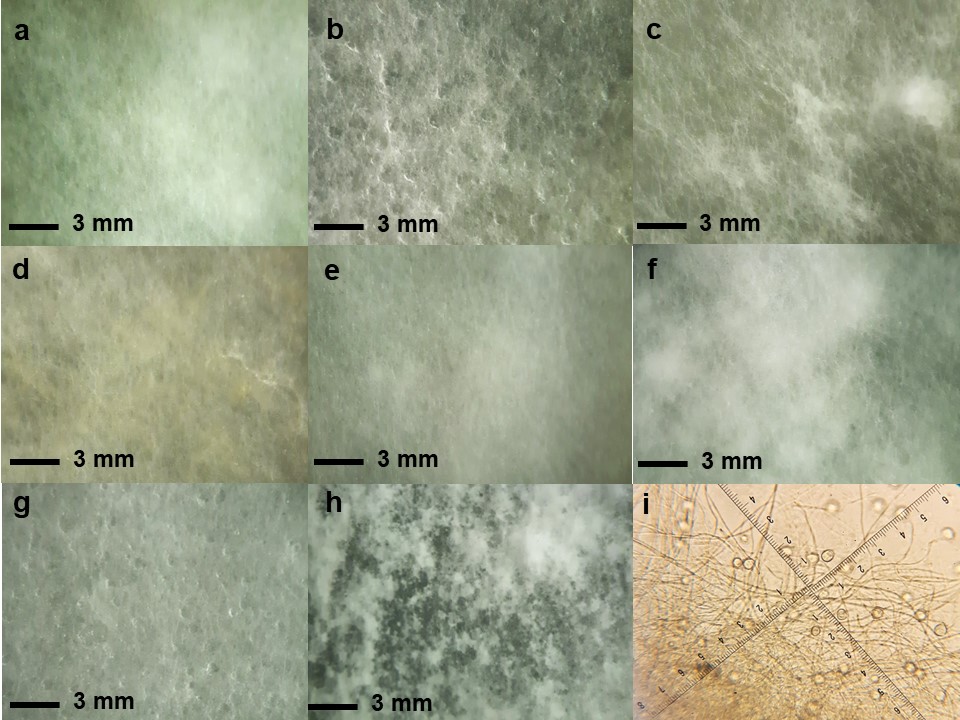

Supplement: Supplementary file 1 [file jof-09-00210-s001.zip › jof-2130688-supplementary.jpg]
